# Asymmetric Membranes Based on Copolyheteroarylenes with Imide, Biquinoline, and Oxazinone Units: Formation and Characterization

**DOI:** 10.3390/polym11101542

**Published:** 2019-09-22

**Authors:** Galina Polotskaya, Alexandra Pulyalina, Mikhail Goikhman, Irina Podeshvo, Iosif Gofman, Sergey Shugurov, Valeriia Rostovtseva, Ilya Faykov, Maksim Tataurov, Alexander Toikka, Alexander Polotsky

**Affiliations:** 1 Saint Petersburg State University, Institute of Chemistry, Universitetskiy pr. 26, Saint Petersburg 198504, Russia; g_polotskaya@mail.ru (G.P.); s.shugurov@spbu.ru (S.S.); st017536@student.spbu.ru (V.R.); st022544@student.spbu.ru (I.F.); st022543@student.spbu.ru (M.T.); a.toikka@spbu.ru (A.T.); 2 Institute of Macromolecular Compounds, Russian Academy of Sciences, Bolshoy pr. 31, Saint Petersburg 199004, Russia; goikhman@hq.macro.ru (M.G.); podeshvo@hq.macro.ru (I.P.); gofman@imc.macro.ru (I.G.); 3 Institute of Highly Pure Biopreparations, Pudozhskaya str. 7, Saint Petersburg 197110, Russia; a.e.polotsky@hpb.spb.ru

**Keywords:** copolyheteroarylene, membrane, ultrafiltration, protein calibration, rejection

## Abstract

Modern ultrafiltration requires novel perfect membranes with narrow pore size, high porosity, and minimal pore tortuosity to achieve high separation performance. In this work, copolyamic acid (co-PAA) was synthesized and used for the preparation of asymmetric porous membranes by phase inversion technique. Several co-PAA membranes were heated up to 250 °C; during heating, they undergo solid-phase transformation into co-polybenzoxazinoneimide (co-PBOI) via dehydration and cyclization. Comparative characterization of both co-PAA and co-PBOI membranes was realized by scanning electron microscopy, mechanical testing, thermogravimetric analysis, and ultrafiltration experiments. Membrane calibration was carried out using a mixture of seven proteins with different molecular weights. During heat treatment, the molecular weight cut-off of the membranes decreased from 20 × 10^3^ g/mol (co-PAA) to 3 × 10^3^ g/mol (co-PBOI). Abnormally low dispersions of rejection (0.3 for co-PAA and 0.45 for co-PBOI) were observed for the studied membranes; this fact indicates that the membranes possess enhanced resolving power.

## 1. Introduction

Pressure-driven membrane technology of ultrafiltration is commercially established for many concentration, fractionation, and purification processes in various industrial areas because of its scalability and efficiency [1,2,3,4]. Membrane ultrafiltration is ubiquitous in many processing industries such as the biotechnology, pharmaceuticals, food and beverage, and paint manufacturing [5,6,7,8,9,10,11,12]. Large scale processes, such as high temperature filtration of viscous oils and aggressive fluids, lignin purification, water purification in nuclear reactors, chemical catalysis, gas phase reactions, etc., need ultrafiltration membranes that exhibit not only high permeability, selectivity, and enhanced mechanical properties but also thermal stability and chemical resistance [13,14,15,16].

The ultrafiltration technology exploits finely porous membranes to retain fine and colloidal impurities, as well as macromolecules with the low molecular weights [17]. The following strategy leads to a high separation performance: Porous membranes with narrow pore size distribution, high porosity, and minimal tortuosity. Asymmetric polymer membranes are traditionally used in ultrafiltration processes. These membranes are obtained by the phase inversion process, when a homogeneous polymer solution is transformed into an anisotropic three-dimensional network structure consisting of a solid polymer frame and voids. The asymmetric membranes are flat films or hollow fibers. Typical parameters of membranes applied for ultrafiltration processes are as follows: Pure water flux (2–500) × 10^−6^ m/(s·bar) in the pressure range of 0.2–3.0 bar, and molecular weight cut-off (1–500) × 10^3^ g/mol [18]. One of the most actual objectives is the development of improved or novel polymer membranes for the effective ultrafiltration processes [19,20].

Polyheteroarylenes are highly demanded materials for membrane technologies, primarily because of their high selectivity, chemical resistance, and thermal stability [13,14,15,16]. Asymmetric ultraporous membranes based on commercially available polyimides, which are soluble in aprotic amide solvents (Matrimid 5218 and P84 copolyimide) [21,22], and series of their modern analogs as aromatic zwitterionic polyimide copolymer [23] are currently known. Such membranes are resistant to many solvents and can be used at elevated temperatures (up to 200 °C) for a long time. However, they are destroyed in the environments containing chlorinated hydrocarbon and amide solvents.

The most chemically resistant and thermally stable polyimide ultrafiltration membranes composed of insoluble and infusible polyimides that are resistant to all organic solvents including amide solvents can be obtained by the two-step method only from their soluble prepolymers (polyamic acids) [13,14]. For example, the asymmetric ultrafiltration membrane based on commercially available poly[(4,4′-oxydiphenylene) pyromelliteimide] has been produced by the phase inversion technique from prepolymer casting solution. After formation of the film, the prepolymer membrane was transformed in solid phase into the polyimide insoluble form at 350 °C. To facilitate conversion of the prepolymer into polyimide and to decrease the conversion temperature down to 200 °C, benzimidazole was used as a catalyst [24,25]; in other work, polyacrylonitrile modified by partial cyclization was used as a promoter, catalyst, and membrane component [26]. Formation of this type of polyimide membrane requires catalytic acceleration, since the prepolymer membrane obtained in the first stage is hydrolytically unstable, i.e., it decomposes quickly upon contact with moisture.

In recent years, a new representative of thermally stable and chemically resistant polyheteroarylenes, namely, polybenzoxazinoneimide (PBOI) has been studied as a membrane material. PBOI combines the valuable performance qualities of two classes of polyheteroarylenes: Polyimides and polybenzoxazinones [27]. One significant advantage of the PBOI prepolymer (imide-containing polyamic acid) is its high hydrolytic stability as compared to that of polyimide prepolymer. PBOIs and their three prepolymers of different structures have been investigated as nonporous films only for gas separation and pervaporation [28,29,30]. It has been shown that the composite membranes including the selective layer of the PBOI prepolymer possess effective dehydrating properties in the pervaporation of water–organic mixtures; besides, they are highly selective in separating industrially important O_2_/N_2_ and H_2_/N_2_ gas pairs. The following fact was established when working with monolithic films of PBOI copolymer: The cyclization of its prepolymer (that has a disordered structure) gives a wider distribution of free volume in the membrane than that in the case of the homopolymer [28].

For the first time in the present work, the copolymer of PBOI (co-PBOI) and its prepolymer (co-polyamic acid (co-PAA)) were selected and investigated as materials for ultrafiltration asymmetric membranes. Synthesis of these polyheteroarylenes includes the following two stages: 1) Polycondensation is used to obtain co-PAA containing preformed imide and biquinoline cycles in the elementary units; 2) thermal treatment leading to dehydration and cyclization processes that results in the transformation of co-PAA into co-PBOI. The co-PBOI chain consists of three types of units: Imide units that provide thermal stability and high strength of membranes, biquinoline units responsible for morphology, and benzoxazinone units. The originality of this study is connected with the high hydrolytic stability of the co-PAA, since it is possible to consider and compare both co-PAA and the product of its cyclization (co-PBOI) as ultrafiltration membranes.

The tasks of this work included the synthesis of the co-PAA copolymer, the formation of asymmetric membranes, the subsequent solid-phase transformation of several co-PAA membranes into co-PBOI, as well as the comparative studies of the structures, thermo-mechanical, and transport ultrafiltration properties of both membranes.

## 2. Materials and Methods 

### 2.1. Materials

**Figure 1:**
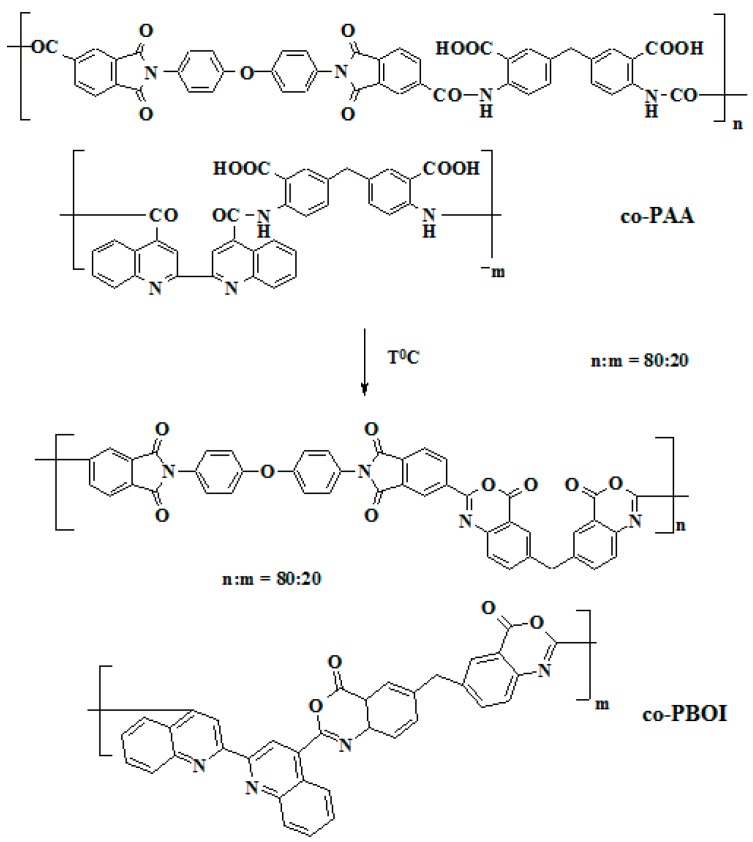
Transformation of the co-PAA into the co-PBOI.

Purification of monomers and solvents, and syntheses of co-PAA (Figure 1) were carried out by techniques described in [27]. Methylenebis (anthranilic acid) (Merck, Darmstadt, Germany) was used without additional purification. Before experiments, thionyl chloride (Vekton, Saint Petersburg, Russia) was distilled, and the fraction boiling at 75.5 °C was collected. *N*-Methylpyrrolidone (NMP) (Vekton, Saint Petersburg, Russia) was also dried over calcium hydride and distilled; *b_p_* = 78 °C at 10 mmHg.

2,2′-biquinoline-4,4′-dicarboxylic acid was synthesized from isatin and acetone (Vekton, Saint Petersburg, Russia) by the Pfitzinger reaction as shown in [31].

#### 2.1.1. Synthesis of Dichloroanhydride of 2,2′-biquinoline-4,4′-dicarboxylic Acid 

The solution of 2,2′-biquinoline-4,4′-dicarboxylic acid (0.016 mol) and thionyl chloride (250 mL) was prepared in a single-neck round-bottom flask equipped with a reflux condenser. The mixture was boiled for 4.5 h.

The crystals precipitated after cooling of the solution were then filtered, washed with toluene, and dried. The yield was 93%. The synthesized dichloroanhydride melts at 251–252 °C.

The syntheses of *N,N*-diphenyl oxide-bis(trimellitimido) acid and its dichloroanhydride were carried out according to the technique described in details in [31].

#### 2.1.2. Synthesis of co-PAA 

Methylenebis (anthranilic acid; 0.002 mol) and NMP (6.5 mL) were mixed in a two-neck flask equipped with a stirrer; stirring was continued until complete dissolution of the acid was achieved.

After the solution was cooled down to −15 °C, the dichloroanhydride of 2,2′-biquinoline-4,4′ -dicarboxylic acid (0.0004 mol) and the dichloroanhydride of *N,N*-diphenyl oxide-bis(trimellitimido) acid (0.0016 mol) were added. The suspension was stirred for 50 min at –15 °C. Then propylene oxide (0.05 mL) was added at the room temperature and stirred additionally for 4–5 h. The resulting co-PAA transparent solution was filtered, degassed, and used for membrane preparation.

### 2.2. Membranes Preparation

#### 2.2.1. Asymmetric Membranes

The co-PAA asymmetric membranes for ultrafiltration (UF) were prepared by the phase inversion technique, i.e., by immersion precipitation. The co-PAA concentration (15, 10, and 8 wt.%) in the casting solution was regulated by dilution with NMP. The co-PAA casting solution was poured onto a glass plate using a casting knife with a nominal thickness of 0.3–0.4 mm, and the glass plate was immersed immediately into a coagulating bath with the water/ethanol mixture containing 40 wt.% ethanol at room temperature. Asymmetric porous membrane was formed as a result of the phase inversion process and kept in the precipitator for ~2 h. Then the membrane was washed with an aqueous solution of ethanol and dried.

The co-PBOI asymmetric membrane was obtained by thermal treatment of the co-PAA membrane formed on a glass plate and fixed on this plate. The co-PAA membrane was heated in a stepwise manner: At 120 °C for 30 min; at 140 °C for 20 min; at 160 °C for 20 min; at 180 °C for 20 min; at 200 °C for 20 min; and at 250 °C for 30 min in the electrical furnace “SNOL 7.2/1100, Lithuania” (Umega Group, Ukmergė, Lithuania) in an argon atmosphere. Transformation of the co-PAA chemical structure into the co-PBOI is shown in Figure 1.

#### 2.2.2. Dense Films 

Dense co-PAA films were prepared by casting a 10 wt.% co-PAA solution in NMP on a glass plate, followed by evaporation of the solvent at 80 °C in air. Films fixed on the glass plate were dried to a constant weight at 80 °C in vacuum for 10 days.

Dense co-PBOI films were obtained by heating the co-PAA films fixed on the glass plate, as a result of reaction (Figure 1). The heating was carried out by a stepwise mode described above.

It should be noted that these dense films were used for IR, spectrometric investigation, and contact angles measurements.

### 2.3. Membrane Characterization

**Figure 2:**
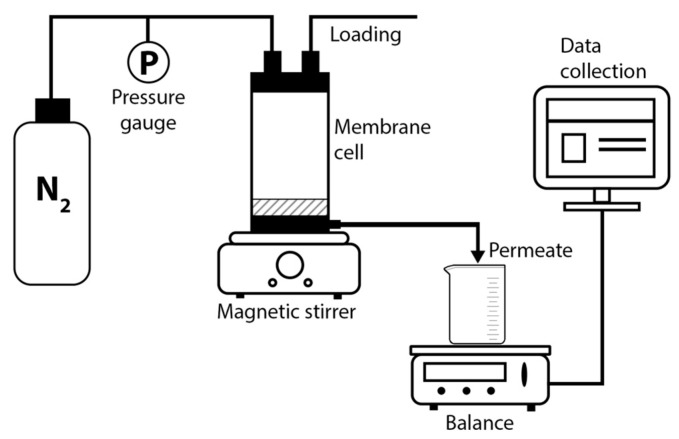
Scheme of the laboratory ultrafiltration setup.

Ultrafiltration tests were carried out in the FM-01 dead-end stirred cell (membrane diameter 25 mm) at room temperature, and at a transmembrane pressure of ~1 bar. Figure 2 shows the scheme of the laboratory ultrafiltration setup. Pressure is provided by nitrogen flow through the top of the cell. Amount of permeate (filtrate) was determined using electronic balance.

The pure water flux {$J_{0}$*,* m/(s·bar)} was defined as the volume of liquid passing through membrane surface area per unit time; it was calculated by Equation (1):

(1) $$J_{0}=V/A\cdot t\cdot \Delta p,$$

where *V* is the volume of the permeate, m^3^; *A* is the membrane surface area, m^2^; *t* is the filtration time, s; and Δp is the transmembrane pressure, bar.

Separation efficiency of the membrane was determined in ultrafiltration experiments using an aqueous solution of 0.4 wt.% model mixture containing proteins with different molecular weights (Table 1) according to the technique described as the calibration procedure in [18].

The composition of the protein mixture in the feed and permeate was analyzed using high pressure exclusion chromatography (GPC-IR, Polymer Char, Valencia, Spain). Chromatographic data were used to calculate the rejection (R) by the Equation (2):

(2) $$R=1-C_{p}/C_{c},$$

where $C_{p}$ is the protein concentration in the filtrate and $C_{c}$ is the protein concentration in the feed, respectively [32].

After the membrane calibration with a model protein mixture, a fouling layer may form on the membrane surface as a result of protein adsorption, which reduces flux through membrane. Therefore, the membranes were washed in an ultrafiltration cell with a phosphate buffer for 10 min and distilled water for 10 min under stirring. Then water flux (*J*_0*t*_) was measured again and flux recovery ratio (*FRR*) was calculated using Equation (3):

(3) $$FRR=J_{0t}/J_{0},$$

where $J_{0}$ is the pure water flux through the membrane, $J_{0t}$ is the water flux after the membrane calibration and washing under stirring in phosphate buffer and distilled water at the same pressure.

Thermogravimetric analysis (TGA) was conducted using a DTG-60 thermal analyzer (Shimadzu, Kyoto, Japan). The samples (~5 mg) were heated up to 600 °C with a scan step of 5 °C /min in air. The TGA curves obtained were used to determine the thermal stability indices of the materials (τ_1_, τ_5_, and τ_10_), i.e., the temperature values at which a polymer loses 1%, 5%, and 10% of its initial weight, respectively, due to the thermal destruction processes.

Mechanical strength of samples was tested with the aid of an AG-100kNX Plus universal mechanical test system (Shimadzu, Kyoto, Japan) using the uniaxial extension mode at room temperature. Strip-like samples with the dimensions 2 mm × 30 mm were stretched at a rate of 10 mm/min, according to ASTM D638 requirements. The Young’s modulus (*E*), the break stress (σ_b_), and the ultimate deformation (*ε*_b_) were calculated using the instrument software.

The glass transition temperature (*T*_g_) was determined using a thermomechanical method at the heating rate of 5 °C/min with the aid of a TMA 402 F1 Hyperion thermal analyzer (NETZSCH, Germany). 

ATR-FTIR spectra of the membranes were recorded on IR-Fourier spectrometer Bruker Tensor 27 (Bruker Daltonics, Billerica, Massachusetts, Germany) with a resolution of 1 cm^−1^ within the range of 4000–500 cm^−1^ at ambient temperature (25 °C).

Mass spectrometric investigation was performed using the Knudsen effusion technique combined with mass spectrometric analysis of the vapor composition with the aid of an MS 1301 mass spectrometer (Construction Department of Russian Academy of Science, Saint Petersburg, Russia). Ionization of the vapor species was carried out by electron ionization, the energy of the ionizing electrons was 25 eV. The samples were evaporated from an open gold effusion cell placed in a molybdenum block and heated using a resistance furnace. The temperature was measured with a Pt−PtRh thermocouple and stabilized with an accuracy of ±1 °C.

Contact angles of liquids on co-PAA and co-PBOI surfaces (in two forms: Dense film and asymmetric UF membrane) were measured via the sessile drop method using the Drop Shape Analyzer DSA 10 (KRÜSS GmbH, Hamburg, Germany) at ambient temperature and atmospheric pressure. Liquids under the study were water and ethylene glycol with surface tension equal to 72.7 mN/m and 47.7 mN/m, correspondingly. For each contact angle measurement, at least five readings from different surface locations were performed, and the results presented are an average of the measured values. The surface tensions ($\sigma_{s}$) were determined by the Owens–Wendt method [33], which allows calculating separately polar ($\sigma^{p}{}_{s}$) and dispersion ($\sigma^{d}{}_{s}$) contributions to the critical surface tension:

(4) $$\sigma_{s}=\sigma^{p}{}_{s}+\sigma^{d}{}_{s}.$$

## 3. Results

Synthesis of co-PAA was carried out by the low-temperature polycondensation. The resulting polymer solution in NMP was used to prepare asymmetric co-PAA membranes by the phase inversion technique. The co-PAA solution deposited on a glass plate after immersion into the water/ethanol (40%) coagulating bath turns into an anisotropic three-dimensional network structure of a solid polymer framework with voids inside. Asymmetric membranes have the form of flat films; membrane formation is a quite technological process due to the high hydrolytic stability of co-PAA. Some of the prepared membranes were converted into asymmetric co-PBOI membranes during step heating up to 250 °C; scheme of the reaction is shown in Figure 1.

**Figure 3:**
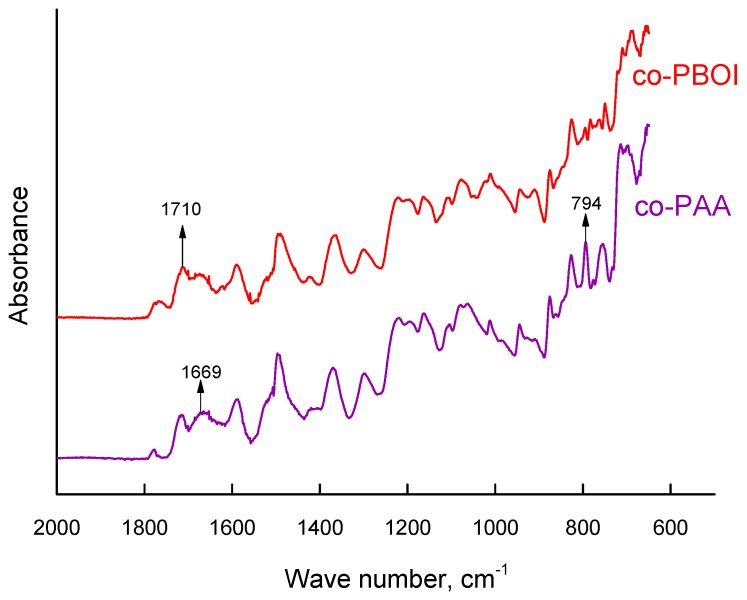
ATR-FTIR spectra of co-PAA and co-PBOI membranes.

The rearrangement of polymer chains was confirmed by IR spectroscopy. Figure 3 shows ATR-FTIR spectra of co-PAA and co-PBOI membranes. Transformation of co-PAA to co-PBOI is accompanied by the appearance of the band at 1710 cm^−1^ corresponding to the carbonyl groups of benzoxazinoneimide [34]. In the co-PAA spectrum, the band at 794 cm^−1^ assigned to the carboxyl group (ν_δ(COO^−^)_) of polyamic acid was apparent as well as the band at 1669 cm^−1^ belonging to the amide group; however, these bands disappeared in the co-PBOI spectrum due to the process of dehydration.

**Figure 4:**
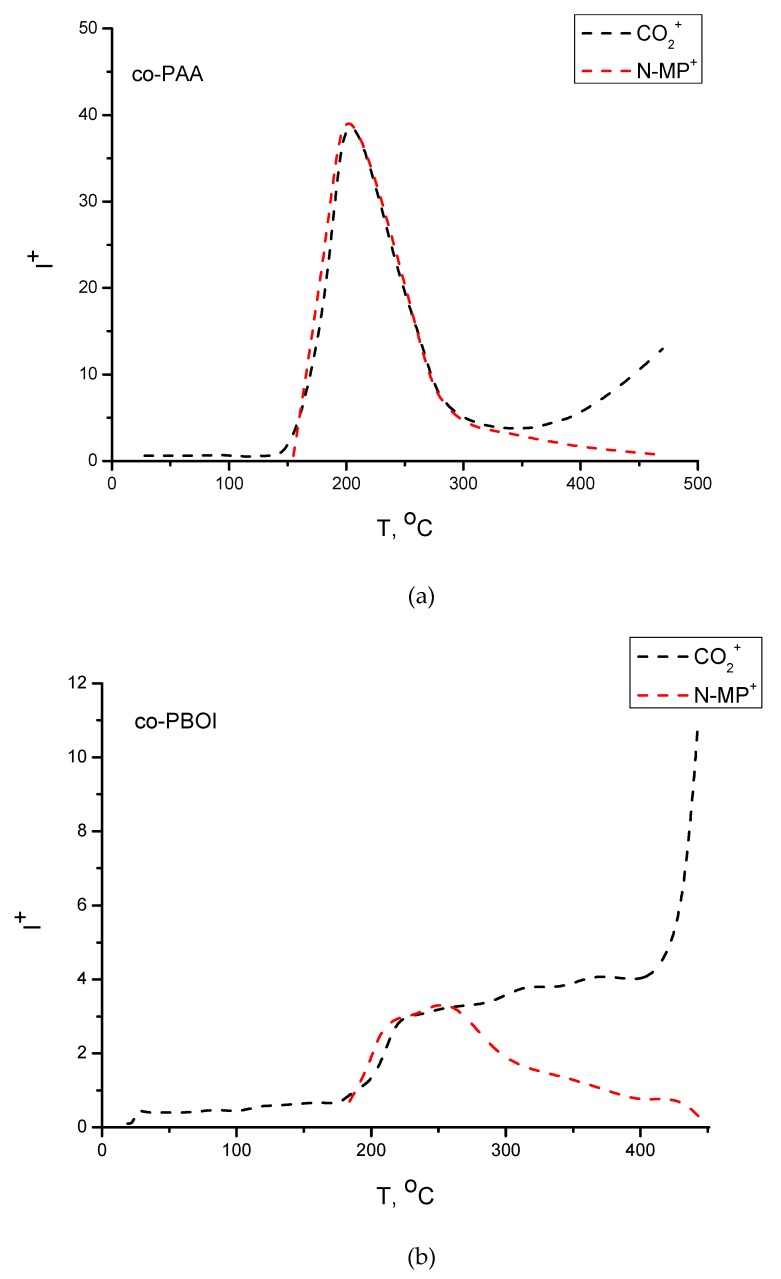
Temperature dependences of intensities of ion currents with m/z = 44 (black dash line, CO_2_^+^ ion) and m/z = 99 (red dash line, NMP^+^ ion) for (**a**) co-PAA and (**b**) co-PBOI.

Mass spectrometric analysis of vapors composition occurring above the co-PAA and co-PBOI films was carried out to investigate the process of heating polymer films. Heating rate was approximately 3.5 K/min. Figure 4 shows temperature dependences of intensities of CO_2_^+^ and NMP^+^ ion currents for the co-PAA and co-PBOI films. There were no other ions (except the atmospheric background (N_2_^+^, O_2_^+^, and Ar^+^)) in the mass spectra. Similar patterns of CO_2_^+^ and NMP^+^ ion currents were observed in the case of co-PAA film. The peak with m/z = 99 corresponds to direct ionization of NMP, whilst the peak with m/z = 44 corresponds to dissociative ionization of NMP or direct ionization of CO_2_ molecules that come from various sources (such as the atmospheric background, and thermal destruction of polymer film). The evaporation of NMP in registerable amounts began at ~155 °C and 184 °C for co-PAA and co-PBOI, respectively. 

At the beginning of the experiment (up to 184 °C in the case of co-PBOI and 155 °C for co-PAA) only CO_2_^+^ ions were detected in mass spectra; these particles appeared as a result of ionization of atmospheric carbon dioxide. The CO_2_^+^ ion current registered in the range from 184 °C to 310 °C origins from atmospheric background and from dissociative ionization of NMP. When the NMP^+^ ion current started decreasing, the CO_2_^+^ ion current increased, because now it came from all three sources. Both polymers demonstrated high thermal stability.

### 3.1. Membrane Morphology

Ultrafiltration membranes are anisotropic structures; the membrane consists of a surface layer with fine pores supported by a much more open microporous substrate. The finely porous surface layer performs the separation; the microporous substrate provides mechanical strength [32]. 

**Figure 5:**
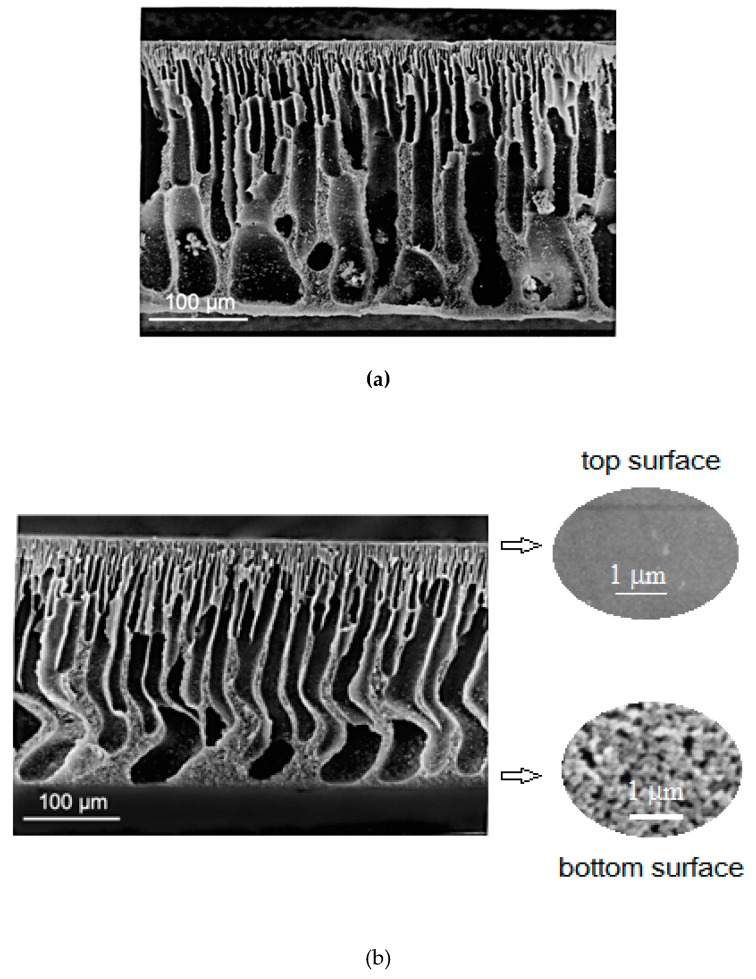
SEM micrographs of membrane cross-section: (**a**) co-PAA, and (**b**) co-PBOI with a detailed view of the top and bottom surfaces.

The morphology of the prepared ultrafiltration asymmetric membranes was studied by scanning electron microscopy. Figure 5 shows micrographs of co-PAA and co-PBOI membrane cross-sections. Detailed view of top and bottom surfaces of the co-PBOI membrane (Figure 5b) demonstrated difference between structures of a finely porous top layer and microporous bottom surface. According to the SEM data, both membranes exhibited high porosity and low tortuosity. The characters of the porous structures of co-PAA and co-PBOI membranes differed slightly. The pores of the co-PAA membrane had an explicit finger-like structure of pores. After heating the film up to 250 °C and its transformation into the co-PBOI membrane, the structure of the finger-like pores was preserved, however, pores became somewhat narrower near the surface layer, and the total thickness of the membrane decreased slightly. 

### 3.2. Mechanical and Physicochemical Properties

**Figure 6:**
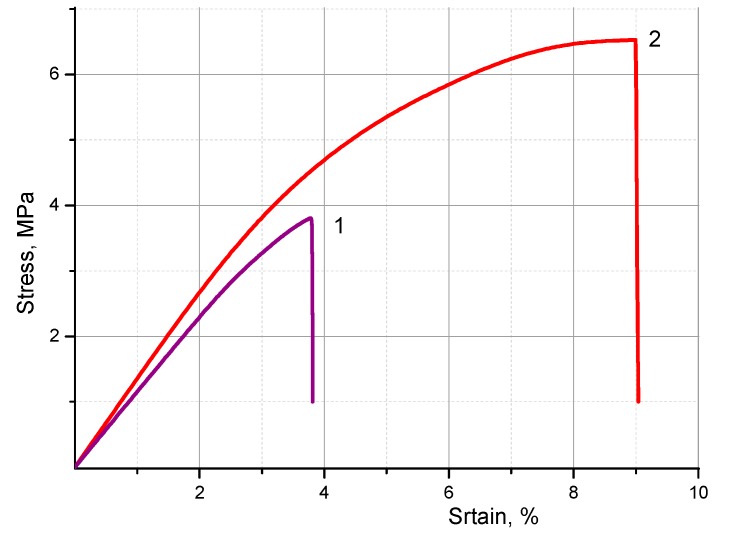
Stress–strain curves of (**1**) co-PAA and (**2**) co-PBOI membrane films.

The results of the mechanical tests of co-PAA and co-PBOI asymmetric membranes (Table 2, Figure 6) testified that the mechanical properties of both materials were suitable for their practical use. To evaluate adequately the values of both the Young’s modulus (*E*) and strength (σ_b_) presented in Table 2, one should take into account the method used for calculating these parameters. Really, these values were calculated without considering the true porosity of the samples, i.e., the calculations were performed for the monolithic films of the same cross-section. While calculating the same parameters for the true polymer part of cross-section, the appropriate values could be obtained that will be several times higher than those listed in Table 2.

To evaluate the impact of the morphology peculiarities on mechanical properties of co-PAA and co-PBOI asymmetric membranes, the SEM micrographs of their cross-sections should be analyzed. Figure 5 demonstrates the developed system of pores that traverse the film in the directions perpendicular to the film surface and hence to the direction of its extension during the mechanical test. In the process of the membrane extension, these pores behaved as the transverse cracks preformed in the bulk of the material. Indeed, these elements of the material structure could be considered as the dominant factor in the formation of the set of its mechanical properties. Destruction of the material started from the very beginning of its extension. This is the only reason that explains the modest values of ultimate deformation registered during the tests (Table 2). These values were substantially lower than those obtained in our previous tests of monolithic co-PBOI and co-PAA film samples of the same composition [29].

The data presented in Table 2 indicate that the transformation of co-PAA membrane into co-PBOI resulted in a substantial (2.5 times) increase in the ultimate deformation and, consequently, in the strength of the material. This effect was caused by changes in morphology of the material that occurred during co-PAA heating, i.e., an increase of the film density and decrease in the characteristic pore size (see the cross-sections of co-PAA and co-PBOI in Figure 5). This suggestion is in good agreement with the fact of decrease in the membrane permeability caused by heating co-PAA and its transformation into co-PBOI, which was registered while studying the transport properties of these materials.

**Figure 7:**
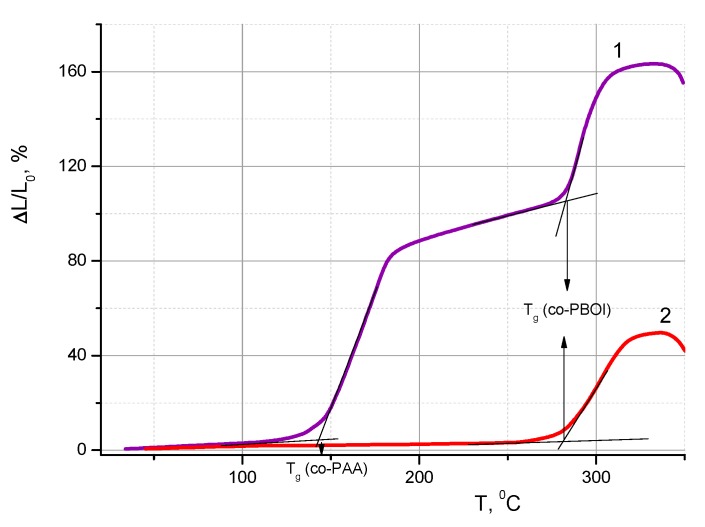
Thermomechanical curves of (**1**) co-PAA and (**2**) co-PBOI membrane films.

The thermomechanical curve of the co-PAA film (Figure 7, curve 1) agreed well with the data obtained earlier in the studies of kinetics of the PAA curing process and formation of PBOI [27]. The conversion process in PAAs of this type began at rather high temperatures (185–190 °C). This characteristic feature of the process made it possible to determine the *T*_g_ values of both co-PAA (before the beginning of curing) and co-PBOI (after the completion of this process) while heating the same sample (Figure 7, curve 1). Obviously, the latter temperature coincided with the *T*_g_ value obtained in the tests involving co-PBOI film (Figure 7, curve 2).

**Figure 8:**
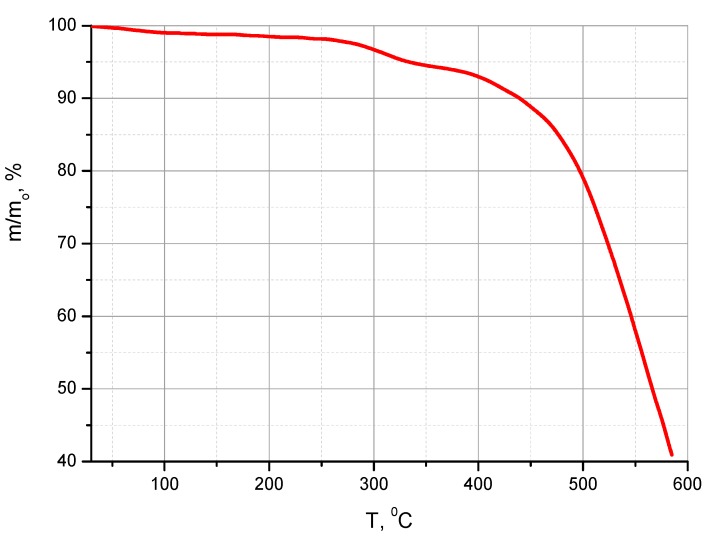
TGA curve of the co-PBOI membrane film.

According to TGA data (Figure 8, Table 2) the co-PBOI was a thermally stable polymer material. During the sample’s heating in TGA chamber, a slight decrease in weight (~4%) was detected in the temperature range of 250–350 °C (which was above the final temperature of co-PAA thermal curing). This decrease in weight occurred at the expense of volatilization of residual NMP, while polymer destruction began at the temperatures exceeding 380–400 °C (Figure 8). Table 2 lists the indices of the thermal stability τ_1_, τ_5_, and τ_10_ corresponding to the temperatures at which the polymer sample loses 1%, 5%, and 10 wt.% of its initial weight. The thermal stability indices of co-PBOI asymmetric membranes were close to those of the previously tested monolithic films of homo-PBOI containing no biquinoline units [27]. The thermal stability indices of co-PAA membrane are not listed in Table 2 because during the heating in the TGA thermal chamber this membrane undergoes the cyclization and forms PBOI. This process terminated at the temperatures that were substantially lower than those of thermal destruction of the material. Therefore, while testing both co-PAA and co-PBOI membranes, the thermal destruction of the same materials was studied.

The most widespread characteristics of solid surfaces are contact angles that allow us to determine surface tension and hydrophilic/hydrophobic capacity of membranes. Contact angles of water and ethylene glycol were measured on the surfaces of dense films and asymmetric UF membranes of co-PAA and co-PBOI (Table 3). The presence of porous layer in UF membranes led to a decrease in contact angle values compared to the dense samples, which was due to the large specific area provided by the porosity and roughness [35]. As seen from Table 3, transformation of co-PAA to co-PBOI increased contact angles of both dense and porous membranes indicating the lower hydrophilic properties of co-PBOI. 

Data on contact angles for dense membranes were used to determine the polar ($\sigma^{p}{}_{s}$) and dispersive ($\sigma^{d}{}_{s}$) contributions to total surface tension ($\sigma_{s}$) by the Owens–Wendt method [33] (Table 3). The polar contribution decreases during the transition from co-PAA to co-PBOI because of cyclization and transformation of amino acid groups into oxazinone rings. The increase in dispersive contribution for co-PBOI can be attributed to the rearrangement of macromolecules in the surface layer. The total critical surface tension for co-PBOI membrane was higher than that for co-PAA. Thus, co-PBOI exhibits more hydrophobic membrane surface as compared with co-PAA. 

### 3.3. Functional Properties of the Ultrafiltration Membranes

Ultrafiltration experiments were carried out to study the permeation and separation performance of co-PAA and co-PBOI asymmetric membranes. Table 4 shows the dependence of pure water flux through the obtained membranes on the polymer (co-PAA) concentration in the casting solution at the membrane formation stage. As expected, an increase in the polymer concentration in the casting solution, significantly reduced the permeability of both membranes. When the casting solution containing 8 wt.% co-PAA was used, membranes with high pure water flux were formed; however, after thermal transformation into co-PBOI, mechanical defects in the membrane porous structure hindered the permeability measurement. The membranes with reliable structures were formed when the concentration of co-PAA in casting solution was equal to 10–15 wt.%. Thermal transformation of the co-PAA membrane into co-PBOI led to the decrease in pure water flux, obviously, due to a regular decrease in the pore sizes of the selective layer.

Another important factor that caused reducing water flux through the membrane was a decrease in hydrophilicity after transformation of co-PAA membrane into co-PBOI. Therefore, it was possible to consider that a decrease in pure water flux through co-PBOI membrane was the result of decrease in membrane hydrophilicity.

**Figure 9:**
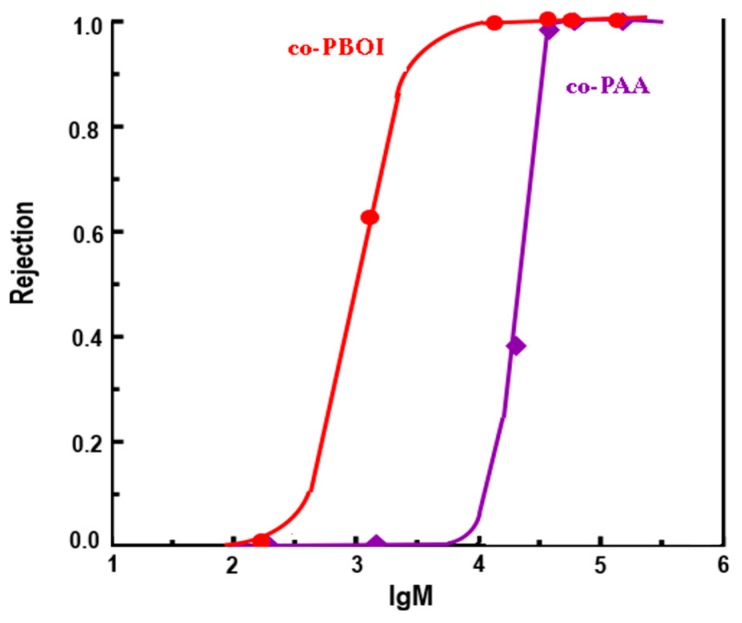
Dependence of proteins rejection on their molecular weights in ultrafiltration using co-PAA and co-PBOI membranes.

Calibration of the membranes with the use of model mixture containing proteins with different molecular weights was made to begin a rapid analysis of ultrafiltration membrane as described by A.N. Cherkasov [18]. Figure 9 shows the rejection curves, which are dependences of protein rejection on protein molecular weights, obtained for co-PAA and co-PBOI membranes.

Data of Figure 9 allowed us to determine two important characteristics of the membrane: i) The molecular weight cut-off (MWCO), i.e., the nominal molecular weight of rejection and ii) the dispersion of rejection. The MWCO value is equal to the molecular weight of a protein rejected by the membrane for not less than 90%. The dispersion of rejection (σ) is calculated by Equation (5):

(5) $$\sigma =0.895\cdot \log\left(M_{0.9}/M_{0.1}\right),$$

where $M_{0.9}$ and $M_{0.1}$ are the molecular weights of the proteins that were rejected by the membrane on 90% and 10%, respectively.

It was found that co-PAA and co-PBOI membranes exhibited good qualities. The initially prepared co-PAA membrane had an MWCO equal to 20 × 10^3^ g/mol, which decreased to 3 × 10^3^ g/mol in cyclized co-PBOI; this fact indicates a decrease of the pore size in the selective layer during the cyclization [36]. The values of dispersion σ calculated according to Equation (5) and Figure 4 were equal to 0.3 (co-PAA) and 0.45 (co-PBOI).

It should be specially noted that co-PAA and co-PBOI membranes were characterized by abnormally low dispersions of rejection, which indicates their enhanced resolution [37]. From our view point, this fact could be explained by the narrow pore size distributions in the selective layers of the investigated membranes. As a rule, dispersion of rejection for standard polymer membranes lies in the range of 0.7–1.5; such membranes have low resolution, i.e., they can completely separate components with a molecular weights differing by tens and hundreds of times. On the contrary, our membrane with dispersion of 0.3 could almost completely separate components with molecular weights differing by less than four times. The investigated co-PAA and co-PBOI membranes were on the way to the perfect ultrafiltration membranes.

In the course of membrane calibration involving a protein mixture, one may meet with the phenomenon of fouling, which is caused by the protein adsorption and cake formation on the membrane surface. This was the reason for the decrease in membrane performance. Fouling of the ultrafiltration membranes can be described by the flux recovery ratio (FRR) [38]. For the membranes under the study the FRR was calculated using Equation (3). It was found that co-PBOI membrane exhibited a high FRR equal to 0.87, while the co-PAA membrane exhibited a common FRR equal to 0.64. A relatively low FRR value indicates higher protein sorption on the surface of the co-PAA membrane. Similar behavior is typical for the majority of known polymer membranes. In the case of co-PBOI membrane, protein sorption is significantly lower in comparison with co-PAA. This may be explained by the fact that carboxyl groups of co-PAA (which can act as sorption sites of proteins) are transformed into oxazinone rings as a result of dehydration during heating. A high FRR magnitude of the co-PBOI membrane is an important performance characteristic, which can facilitate the process of membrane regeneration, as well as reduce the loss of target substances.

**Figure 10:**
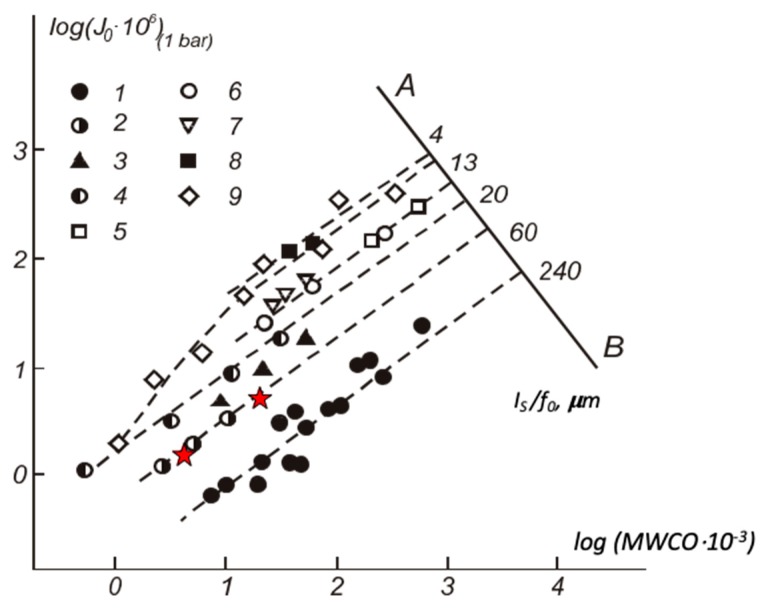
Classification diagram of ultrafiltration membranes [37]: 1, Sartorius SM 115–117, and Vladipore UAM, 2, Biopore A2–10, 3, DDS-5,600, –800, 4, Diaflo UM, 5, Biopore A-20-500, 6, Diaflo XM, 7, Dorr-Oliver XP, 8, Diaflo PM, 9, Gelman Omega. Red stars represent the data obtained for co-PAA (right) and co-PBOI (left) membranes.

Comparison of the novel co-PAA and co-PBOI membranes with the commercial membranes was performed using the universal classification diagram [39], which gives the principal parameters of ultrafiltration membranes, i.e., flux (*J*_0_) and molecular weight cut-off (MWCO; Figure 10). The point of intersection of the dotted line with the AB axis allows determining of the reduced thickness of the selective layer (*l*_S_/*f*_0_), where *l*_S_ is the geometric thickness of the selective layer and *f*_0_ is the porosity of the selective layer. The reduced thickness of the selective layer was about 60 µm for co-PAA and co-PBOI membranes. According to SEM, the geometric thickness of the selective layers of the investigated membranes was about 1 µm, thus, the porosity of their selective layers was in the range of 1–1.5. Figure 10 shows that the performance characteristics of the studied membranes were inferior to the best samples of the commercial membranes. At the same rejections, the pure water fluxes of co-PAA and co-PBOI membranes were lower than that of the commercial membranes. This fact was a consequence of significantly higher values of *l*_S_/*f*_0_ for selective layers of co-PAA and co-PBOI membranes, most likely due to their low porosity. The increase in porosity was possible by changing in conditions of membrane preparation (an inclusion of pore forming agents, etc.), which could lead to a significant improvement of membrane characteristics.

## 4. Conclusions

In the present work, two membranes (co-PAA and co-PBOI) based on copolyheteroarylenes, that contain imide units (which provide thermal stability and high strength of membranes) and biquinoline units (responsible for morphology) were developed in this work. The co-PAA was synthesized by low temperature polycondensation and used for the preparation of asymmetric porous membranes by the phase inversion technique. Several membranes were heated up to 250 °C to transform co-PAA into co-PBOI by dehydration and cyclization that leads to the formation of benzoxazinone units. The rearrangement of polymer chains was confirmed by ATR-FTIR spectra. High hydrolytic stability of co-PAA prepolymer makes the process of the membrane formation technologically feasible. SEM studies on morphology showed that asymmetric co-PAA membranes had a regular structure with thin pores that was preserved after thermal transformation of samples into co-PBOI. Mechanical and thermal tests demonstrated that both membranes possessed suitable operating ability. Measurements of contact angles and calculation of surface tension revealed that the co-PBOI membrane exhibited a more hydrophobic membrane surface as compared with co-PAA.

Ultrafiltration experiments revealed permeation and separation performances of the developed asymmetric membranes. It was found that the pure water flux through the co-PBOI membrane was lower than that through the co-PAA because of the decrease in membrane hydrophilicity and reduction in pore size of the selective layer. Separation efficiency of the membranes was investigated by the calibration of membranes using a mixture of proteins with different molecular weights. It was found that co-PAA and co-PBOI membranes had good qualities. The molecular weight cut-off of the co-PAA membrane (which was equal to 20 × 10^3^ g/mol) decreased down to 3 × 10^3^ g/mol during the heat-induced transformation into co-PBOI, as a result of decreasing pore size in the selective layer during the cyclization. It is important to note that our membranes had abnormally low dispersions of rejection for our membranes equal to 0.3 (co-PAA) and 0.45 (co-PBOI), which indicated their enhanced resolving power. Synthesis of these membranes will make ultrafiltration one of the most highly efficient methods of separating macromolecules according to their sizes and molecular weights.

Chemical structure of the developed copolyheteroarylene membranes containing imide and benzoxazinone units is a guarantee of their promising application in membrane technologies, where the membranes are operated at high temperatures and in aggressive environments.

## Figures and Tables

**Table 1:** Model mixture of proteins for membrane calibration; *M* is the molecular weight of a protein, and *r_S_* is the Stokes radius of a protein molecule.

No	Component	*M* 10^−3^, g/mol	*r_S_*, Ǻ
1	Tryptophan	0.204	4.8
2	Vitamin B_12_	1.36	7.8
3	Cytochrome C	12.4	17.6
4	Chymotrypsinogen	24.0	22.7
5	Ovalbumin	44.0	28.6
6	Bovine serum albumin	67.0	34.0
7	γ-globulin	160.0	46.5

**Table 2:** Mechanical and thermal properties of asymmetric membranes.

Polymer	*T*_g_, °C	*E*, MPa	σ_b_, MPa	*ε*_b_, %	τ_1_, °C	τ_5_, °C	τ_10_, °C
co-PAA	145	135 ± 8	4.1 ± 0.4	3.8 ± 0.5	-	-	-
co-PBOI	280	151 ± 9	6.7 ± 0.2	9 ± 1	403	448	478

**Table 3:** Contact angles and surface tension of co-PAA and co-PBOI samples.

Sample	Contact angle, °		Surface tension, mJ/m^2^		
	Water	Ethylene glycol	$\sigma^{p}{}_{s}$	$\sigma^{d}{}_{s}$	$\sigma_{s}$
Dense co-PAA	79.1	50.8	7.8	24.7	32.5
Dense co-PBOI	85.1	51.9	2.9	34.0	36.9
UF co-PAA	74.9	36.9			
UF co-PBOI	76.1	38.8			

**Table 4:** The effect of the polymer (co-PAA) concentration in the casting solution on pure water flux (*J*_0_) through membranes.

co-PAA in the casting solution, wt.%	*J*_0_·10^4^, m/(s·bar)	
	co-PAA membrane	co-PBOI membrane
8	317	-
10	140	10
15	4.8	1.1
